# Isolation of a novel orthobunyavirus from bat flies (*Eucampsipoda africana*)

**DOI:** 10.1099/jgv.0.000753

**Published:** 2017-05-11

**Authors:** Petrus Jansen van Vuren, Michael R. Wiley, Gustavo Palacios, Nadia Storm, Wanda Markotter, Monica Birkhead, Alan Kemp, Janusz T. Paweska

**Affiliations:** ^1^​ Centre for Emerging and Zoonotic Diseases, National Institute for Communicable Diseases, National Health Laboratory Service, Sandringham, South Africa; ^2^​ Department of Microbiology and Plant Pathology, Faculty of Natural and Agricultural Science, University of Pretoria, South Africa; ^3^​ Centre for Genome Sciences, United States Army Medical Research Institute of Infectious Diseases, Frederick, MD, USA; ^4^​ Centre for Viral Zoonoses, Department of Medical Virology, Faculty of Health Sciences, University of Pretoria, South Africa; ^5^​ Faculty of Health Sciences, University of the Witwatersrand, Johannesburg, South Africa

**Keywords:** arbovirus, *Rousettus aegyptiacus*, Egyptian fruit bat, *Bunyaviridae*, orthobunyavirus, bat flies, *Eucampsipoda africana*, pathogen discovery

## Abstract

The *Bunyaviridae* family comprises viruses causing diseases of public and veterinary health importance, including viral haemorrhagic and arboviral fevers. We report the isolation, identification and genome characterization of a novel orthobunyavirus, named Wolkberg virus (WBV), from wingless bat fly ectoparasites (*Eucampsipoda africana*) of Egyptian fruit bats (*Rousettus aegyptiacus*) in South Africa. Complete genome sequence data of WBV suggests it is most closely related to two bat viruses (Mojuí dos Campos and Kaeng Khoi viruses) and an arbovirus (Nyando virus) previously shown to infect humans. WBV replicates to high titres in VeroE6 and C6-36 cells, characteristic of mosquito-borne arboviruses. These findings expand our knowledge of the diversity of orthobunyaviruses and their insect vector host range.

## Abbreviations

CPE, cytopathic effect; EMEM, Eagle’s minimum essential medium; KKV, Kaeng Khoi virus; L, large; M, medium; MDCV, Mojuí dos Campos virus; NP, nucleocapsid protein; RdRp, RNA-dependent RNA-polymerase; RER, rough endoplasmic reticulum; S, small; TCMV, Tacaiuma virus; WBV, Wolkberg virus.

## Introduction

The *Bunyaviridae* family contains over 530 members and has an extensive host range, including arthropods, rodents, large mammals and plants. The typical bunyavirus genome comprises single-stranded RNA separated into small (S), medium (M) and large (L) segments with complementary terminal sequences unique to specific viral genera. The majority of bunyavirus genomes are negative sense, but some viruses use ambisense strategies to express genes from the S segment. The S segment encodes the nucleoprotein, the M segment encodes the glycoprotein precursor and the L segment encodes the RNA-dependent RNA polymerase [1, 2].

The orthobunyavirus genus comprises more than 170 known viruses assembled into 48 species and 19 serogroups [2]. Viruses in the genus, such as La Crosse, Ngari, Oropouche and Nyando viruses, are known to cause disease in humans, ranging from mild febrile illness to more severe complications including encephalitis, haemorrhagic fever and death [3–11]. Viruses of veterinary importance include Schmallenberg, Akabane and Shuni viruses [12–14]. Orthobunyaviruses are primarily transmitted by arthropods. Mojuí dos Campos (MDCV) and Kaeng Khoi (KKV) viruses have been isolated from bats in South America and East Asia, respectively, while KKV was also isolated from bedbugs [15–17]. Some serological evidence suggests that KKV might be of public health importance [18].

Several orthobunyaviruses have been detected in South Africa (Shuni, Pongola and Bunyamwera viruses amongst others) [4, 5, 13], but none are known to be associated with bats or their ectoparasites. We describe the discovery of the first orthobunyavirus to be isolated from bat ectoparasites (*Eucampsipoda africana)*, which were collected from wild-caught Egyptian fruit bats (*Rousettus aegyptiacus*) in South Africa. The virus has been named Wolkberg virus (WMV), after the location of the cave harbouring the Egyptian fruit bats from which these ectoparasites were collected.

## Results and discussion

### Virus isolates and growth

**Fig. 1.**
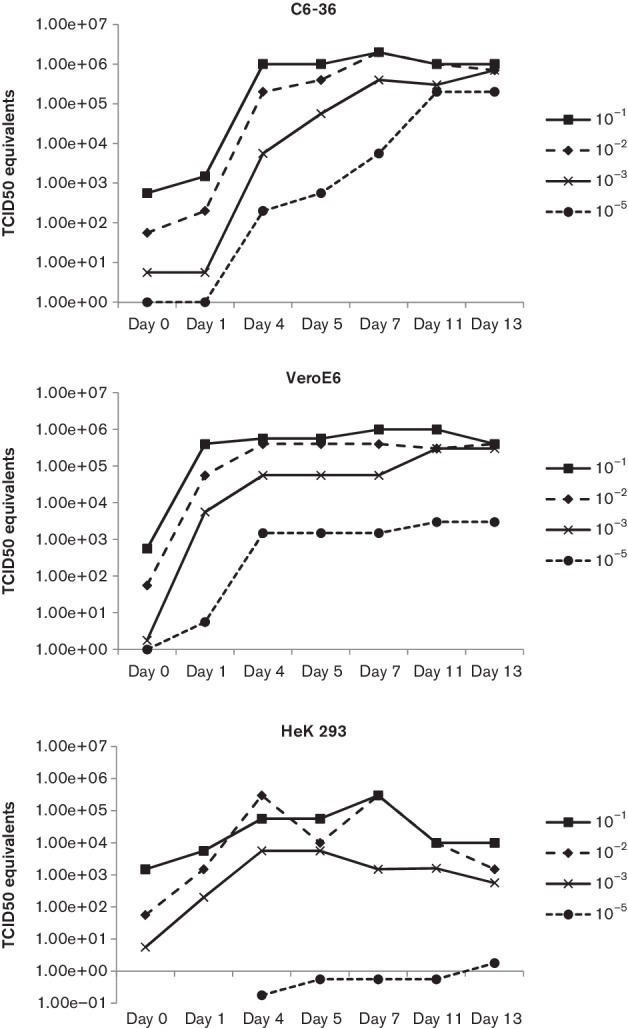
Growth curve of WBV in C6-36, VeroE6 and HEK293 cells. Note: virus present on day 0 is due to inoculum.

From a total of 273 bat ectoparasite pools tested, 11 caused a non-characteristic cytopathic effect (CPE) by day 5–7 post inoculation (from the third passage onwards) in the form of small, rounded, refractory detached cells in the presence of a mostly intact Vero monolayer. All ectoparasite pools originated from apparently healthy Egyptian fruit bats. The 11 isolates replicated to titres in excess of 1×10^5.0^ TCID_50_ ml^−1^ in Vero cells by the third passage. A standard curve (R^2^=0.999) generated by triplicate testing of a dilution series of stock virus enabled us to establish TCID_50_ equivalents relative to RT-PCR *C*_t_ values (Table 1). This enabled us to determine TCID_50_ equivalent values for RT-PCR results obtained in further testing. WBV displayed similar growth curve characteristics in Vero and C6-36 cells over a period of 2 weeks (Fig. 1). However, in HEK293 cells the virus replicated to lower levels relative to Vero and C6-36 cells at the same days post inoculation with the same concentration of stock virus. The difference in yield (TCID_50_ equivalents) between HEK293 and the other cells lines ranged from 1 log to 3 logs in some cases, especially with the lowest virus input. The ectoparasites from which the viruses were isolated were morphologically identified as nycteribiid bat flies, *Eucampsipoda africana* Theodor (*Diptera: Nycteribiidae*) [19, 20].

**Table 1.** Correlation of real-time RT-PCR *C*_t_ values to TCID_50_

Real-time RT-PCR *C*_t_ value	Corresponding TCID_50_ equivalent value
<16	>1×10^6.75^
16–19	1×10^5.75^ to 1×10^6.75^
19–23	1×10^4.75^ to 1×10^5.75^
23–27	1×10^3.75^ to 1×10^4.75^
27–30	1×10^2.75^ to 1×10^3.7^5
30–34	1×10^1.75^ to 1×10^2.75^
34–38	1×10^0.75^ to 1×10^1.75^
38–40	1×10^0.25^ to 1×10^0.75^
40–42	1×10^−0.25^ to 1×10^0.25^
42–45	1×10^−0.75^ to 1×10^−0.25^

**Fig. 2.**
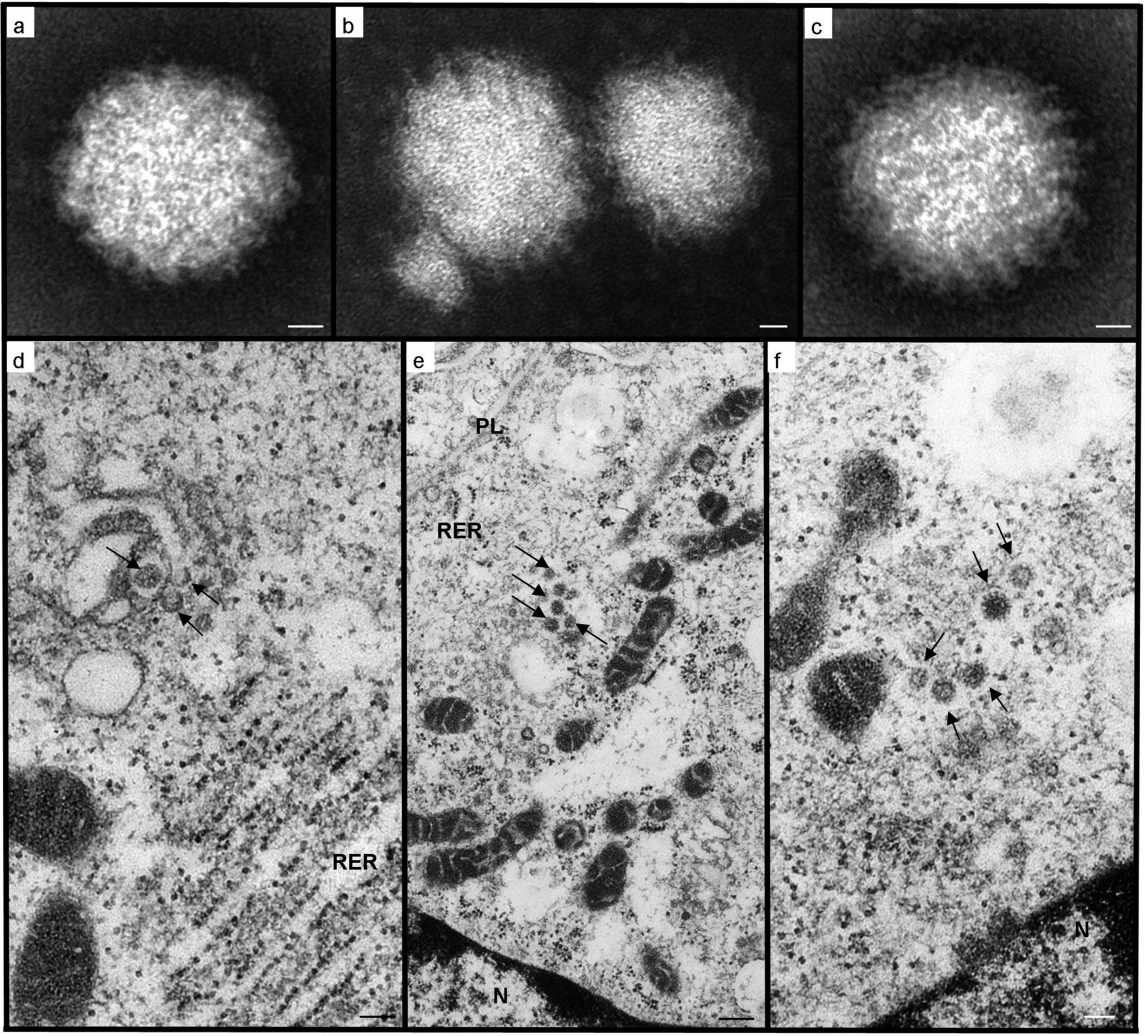
Transmission electron microscopy of WBV negatively stained particles (a, b, c) and infected Vero cells (d, e, f). (a) Virion of icosahedral-spherical shape, with an apparently amorphous envelope; (b) heterogeneity in particle size; (c) virus particle in which regularly spaced, envelope projections (right side) contrast with the fuzzy appearance of the envelope (left side); (d) transverse sections through characteristic tubular elements (arrows) forming within Golgi cisternal stacks, which are in close proximity to mitochondrial profiles and distended rough endoplasmic reticulum (RER); (e) developing virions (arrows) within a Golgi-derived vesicle, approaching the plasmalemma (PL) for exocytosis. Note the organelle arrangement with the nucleus (N) and numerous mitochondrial profiles in juxtaposition to the disrupted Golgi body; (f) mature virions (arrows) with glycoprotein envelope spikes, still within an endomembrane that appears continuous with those of the tangentially sectioned mitochondrial profiles. Scale bars: (a, b, c) 12 nm; (d, f) 100 nm; (e) 250 nm.

Identification by transmission electron microscopy and next-generation-sequencing negatively-stained preparations of culture supernatants revealed numerous enveloped, roughly spherical virions, heterogeneous in size (between 78 and 111 nm), but with an average diameter of 87 nm (*n*=100). The envelope appearance varied from indistinct and fuzzy, to fairly regularly spiked, in which projections measured up to 8 nm (Fig. 2a–c). These features conform to those described for viruses of the *Bunyaviridae* family [2], specifically of the genus *Orthobunyavirus* [21, 22].

This taxonomic assignation was supported by ultrastructural details of infected cells (Fig. 2d–f). Virus particles develop within the Golgi body, initially from round and tubular structures, which to date are unique to *Orthobunyavirus* infections [23]. The Golgi body is located close to one side of the nucleus (frequently within an indentation), and the tubular viral factories are connected to both rough endoplasmic reticulum and numerous mitochondrial profiles, as in Bunyamwera virus morphogenesis [24]. The Golgi body becomes increasingly disrupted, and enveloped particles can be seen within Golgi-derived vesicles being trafficked towards the cell membrane for exocytosis, as described for other orthobunyaviruses [16, 25].

Identification as an orthobunyavirus was confirmed by using an unbiased next-generation sequencing approach that resolves the 5′ and 3′ termini (sequence-independent single-primer amplification combined with rapid amplification of cDNA ends, SISPA-RACE). Assembled contigs were aligned to the nt sequence database (blast, GenBank). All 11 isolates contained contigs that had matches for an S, M and L segment of an orthobunyavirus.

### Genome and phylogenetic analysis

Maximum-likelihood trees, constructed with nucleic acid sequence data for the L, M and S segments of representative viruses from the different genera within the *Bunyaviridae* family (see Figs S1–S3, available in the online Supplementary Material), show the placement of the 11 WBV isolates amongst other orthobunyaviruses in the family. Table 2 provides information on the sequence data obtained from the 11 WBV isolates. All WBV genome segments for which complete sequences were obtained, or at least complete sequences at either terminal end, had identical terminal sequences that were complementary to each other at the 5′ and 3′ ends. These 5′ and 3′ terminal end sequences are 5′-AGTAGTGT and ACACTACT-3′ respectively, in the antigenomic (positive) sense. These terminal complimentary sequences are characteristic of viruses in the *Orthobunyavirus* genus [2].

**Table 2.** Sequence information for the 11 WBV isolates Complete and coding complete (CC) descriptions were defined previously [44]. If genome is coding complete but does have one of the ends complete, 5C or 3C denotes a complete sequence at the 5′ or 3′ end of the segment sequence, respectively.

WBV isolate number	Genome segment	Genome segment length (nucleic acid)	Sequence completeness	ORF length (nucleic acid)	GenBank accession number
2562	L	6873	Complete	6759	KX470551
	M	4461	Complete	4266	KX470552
	S	978	Complete	702	KX470553
2761	L	6873	Complete	6759	KX470554
	M	4471	Complete	4266	KX470555
	S	978	Complete	702	KX470556
2763	L	6800	CC	6759	KX470557
	M	4465	Complete	4263	KX470559
	S	967	CC (5C)	702	KX470558
2795	L	6873	Complete	6759	KX470560
	M	4461	Complete	4266	KX470561
	S	978	Complete	702	KX470562
2812	L	6873	Complete	6759	KX470563
	M	4461	Complete	4266	KX470564
	S	978	Complete	702	KX470565
2813	L	6873	Complete	6759	KX470566
	M	4468	Complete	4266	KX470567
	S	978	Complete	702	KX470568
2818	L	6873	Complete	6759	KX470569
	M	4461	Complete	4266	KX470570
	S	978	Complete	702	KX470571
2824	L	6873	Complete	6759	KX470572
	M	4465	Complete	4263	KX470573
	S	979	Complete	702	KX470574
3011	L	6841	CC (3C)	6759	KX470575
	M	4425	CC (5C)	4266	KX470576
	S	818	CC	702	KX470577
3264	L	6873	Complete	6759	KX470578
	M	4461	Complete	4266	KX470579
	S	978	Complete	702	KX470580
SM910	L	6873	Complete	6759	KX470581
	M	4461	Complete	4266	KX470582
	S	978	Complete	702	KX470583

**Fig. 3.**
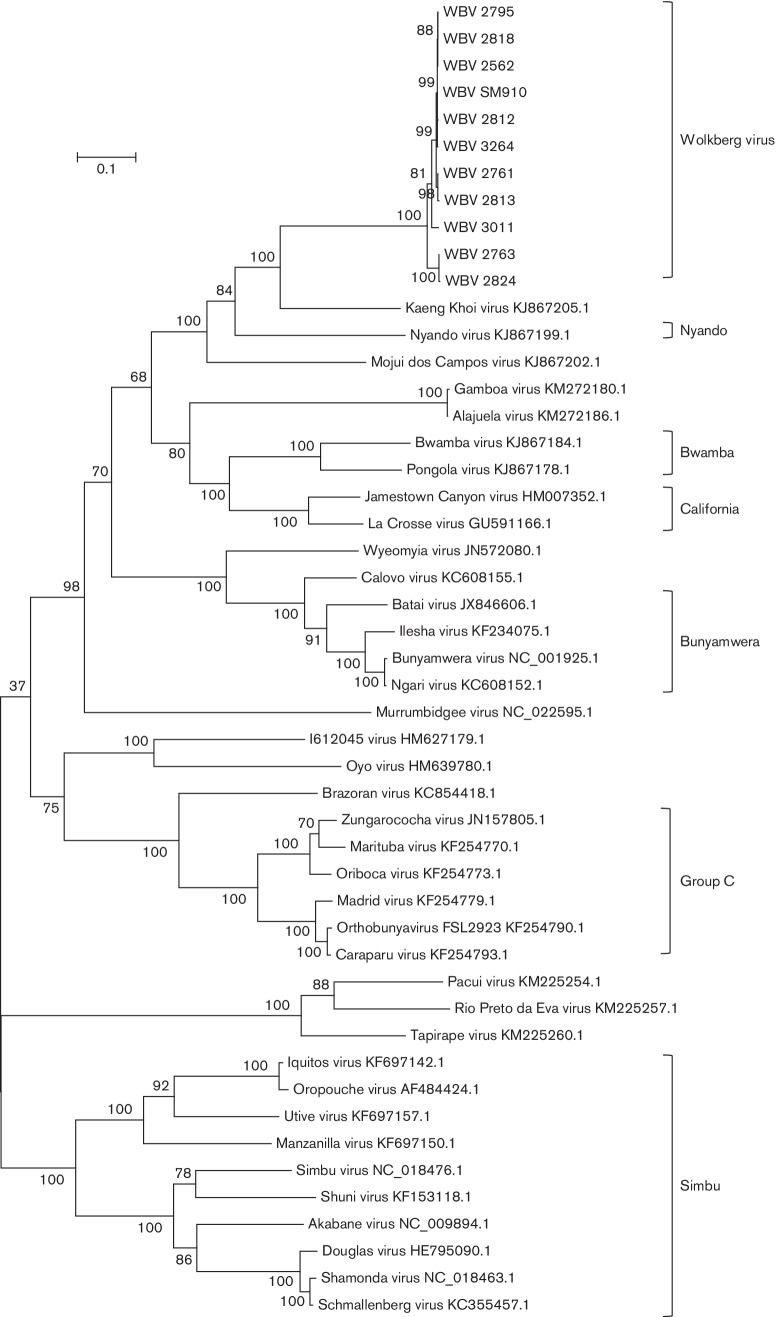
Molecular phylogenetic analysis by the maximum-likelihood method showing representative viruses from different viruses in the *Orthobunyavirus* genus using L-segment ORF amino acid sequences. GenBank accession numbers are indicated next to virus names (excluding WBV for which accession numbers are provided in Table 2). Medically important serogroups, and WBV isolates, are indicated by brackets.

**Fig. 4.**
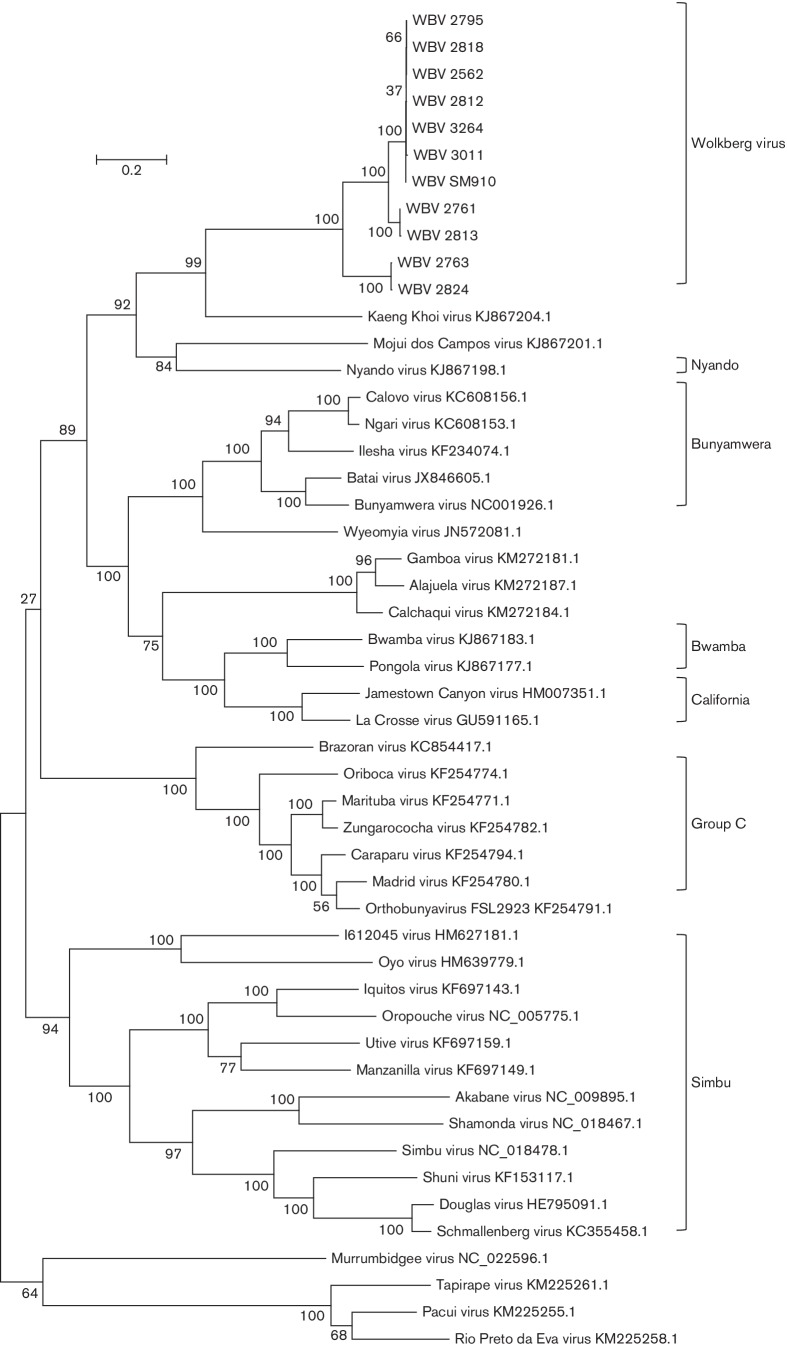
Molecular phylogenetic analysis by the maximum-likelihood method showing representative viruses from different viruses in the *Orthobunyavirus* genus using M-segment ORF amino acid sequences. GenBank accession numbers are indicated next to virus names (excluding WBV for which accession numbers are provided in Table 2). Medically important serogroups, and WBV isolates, are indicated by brackets.

**Fig. 5.**
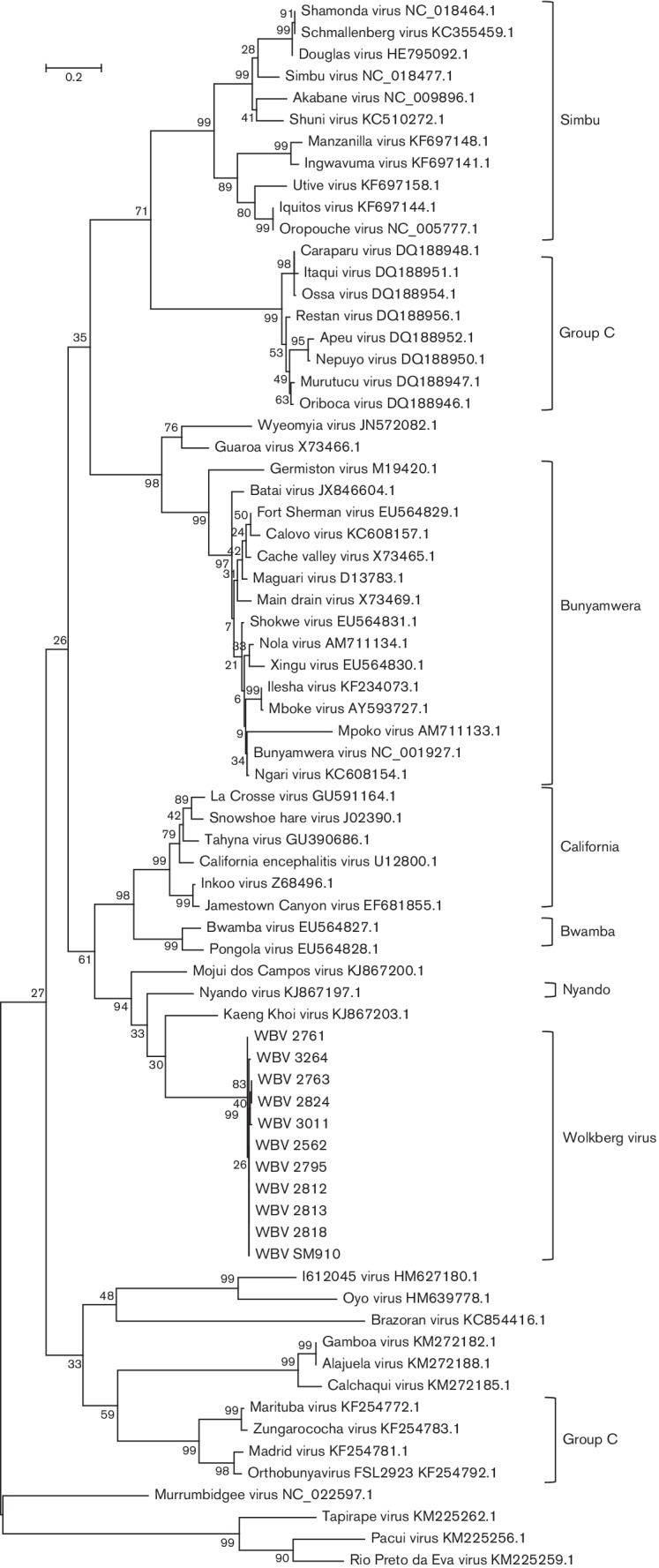
Molecular phylogenetic analysis by the maximum-likelihood method showing representative viruses from different viruses in the *Orthobunyavirus* genus using the S-segment ORF amino acid sequences. GenBank accession numbers are indicated next to virus names (excluding WBV for which accession numbers are provided in Table 2). Medically important serogroups, and WBV isolates, are indicated by brackets.

Maximum-likelihood trees were prepared using the WBV deduced amino acid sequences from the ORFs of the three segments and those of other viruses in the *Orthobunyavirus* genus (Figs 3–5). Only the ORF encoding the nucleocapsid protein was used from the S segment since WBV does not have a second ORF in the S segment. The ORF encoding the Gn-Gc-NSm polyprotein was used from the M segment.

A distinct clade is formed by the 11 WBV isolates, KKV, MDCV and Nyando virus (Figs 3–5) within the *Orthobunyavirus* genus. WBV is most closely related to KKV based on L-, M- and S-segment nucleic acid identity (67.6 %, 56.5 %, 65.5 %) and ORF-deduced amino acid sequences of the L and M segments (66.3 %, 46.4 %), but closest to MDCV based on an S-segment ORF amino acid sequence (65.6 %). The ICTV species demarcation in the *Orthobunyavirus* genus is complicated by the lack of biochemical characterization of named viruses, and species are thus demarcated based on serological cross-reaction criteria [2]. In the absence of these data, another possible criterion is nucleocapsid protein amino acid difference, where differences of more than 10 % represent different species. Based on the fact that the closest-related virus to WBV based on the nucleocapsid protein amino acid sequence differs by 34.4 % (MDCV, based on currently available sequence data), WBV is likely a new species in the *Orthobunyavirus* genus. However, this needs to be further investigated by evaluating serological cross-reactivity to closely related viruses. The sequences of the 11 WBV isolates are not identical. There appear to be three sub-lineages of the virus based on the ORF-deduced amino acid sequences of all three segments (Figs 3–5), more noticeably based on the M-segment ORF amino acid sequence.

Percentage pairwise differences in sequences between the 11 isolates range from being identical (all segments) to 4.1 % (L-segment ORF), 28.8 % (M-segment ORF) or 1.3 % (S-segment ORF). Based on the M-segment ORF, the WBV isolates 2763 and 2824 seem to be closely related (0.4 % p-distance) but quite distinct from the other nine isolates (between 27.9 and 28.8 % difference). The close-relatedness between all 11 isolates is based on L- and S-segment ORFs, but clear divergence in two isolates in the M-segment might represent evidence of re-assortment with another variant of WBV that is yet to be isolated, and thus possibly evidence of higher undiscovered divergence of the new virus. Based on limited data which suggest that re-assortment can only occur between viruses in the same species [2] and the fact that the M-segment ORFs of isolates 2763 and 2824 are no more than 46.5 % similar to any other currently known orthobunyavirus (for which sequence data are available), it is likely that re-assortment occurred with another, yet undiscovered, member of the proposed WBV species in the *Orthobunyavirus* genus.

### RT-PCR detection of WBV in bat fly homogenates and bat serum

The original 11 homogenates from which the strains of WBV virus were isolated were subsequently tested by RT-PCR to determine viral loads. Similarly, bat serum pools from around the period of virus isolation were also tested by RT-PCR. The results are summarised in Table 3. WBV was readily detected, at relatively high concentrations, in bat fly homogenates (9/11). However, WBV was only detected in one serum pool from Egyptian fruit bats (1/85 pools of five bat sera), and at relatively low concentration. Considering the transient viraemia of most arboviral infections, this is not surprising. Detection of the virus in the serum of one bat demonstrates that it is likely that Egyptian fruit bats can be infected by WBV, thereby probably playing a role in its maintenance.

**Table 3.** Detection of WBV in bat fly homogenates and Egyptian fruit bat serum

Bat ID number	Real-time RT-PCR *C*_t_ value	TCID_50_ equivalent ml^−1^
2824	26.37	1×10^3.75^
2763	34.29	1×10^1.75^
2761	25.43	1×10^4.2^
2818	31.7	1×10^2.3^
2795	Neg	N/A
2812	22.81	1×10^4.75^
2813	23.1	1×10^4.75^
2562	Neg	N/A
3011	33.54	1×10^1.9^
SM910	26.42	1×10^3.75^
3264	23.36	1×10^4.8^
5× bat sera/pool (*n*=85)	1 positive (*C*_t_ 38.79)	1×10^0.75^

### ORFs

#### RNA-dependent RNA-polymerase (RdRp)

The deduced 2253 aa size of the WBV RdRp (262 kDa, pI 6.88) corresponds to the size of the same protein of other members of the *Orthobunyavirus* genus (Bunyamwera virus RdRp 2238 aa) [26].

#### Gn-Gc-NSm polyprotein

The 1422 aa WBV polyprotein (162 kDa, pI 8.33) is likely co-translationally cleaved into the 951 aa G1 (108.8 kDa, pI 6.56), 285 aa G2 (32 kDa, pI 8.83) and 166 aa NSm (19 kDa, 9.34). Cleavage of the signal peptide is predicted at position 18 relative to the first methionine (SignalP 3.0). The G2 contains the conserved arginine at position 303, which likely represents the cleavage site from the downstream NSm [27]. SignalP predicts the cleavage site between NSm and the downstream G1 at ILI_470_-EA, which corresponds to other orthobunyaviruses [28]. Six potential glycosylation sites were identified using NetNGlyc 1.0 (www.cbs.dtu.dk/services), of which one is in the G2, three in the NSm and two in the G1 protein.

### Nucleocapsid protein

The WBV-deduced nucleocapsid protein (NP) is 234 aa in length (26.5 kDa, pI 9.44) and corresponds to other orthobunyaviruses [28]. It satisfies the ICTV criterion of >10 % divergence from other viruses in the *Orthobunyavirus* genus to regard it as a separate, new species [2]. The four residues involved in ribonucleoprotein complex formation (P_125_, G_131_, Y_158_ and I_231_) are all conserved in WBV [29]. Contrary to most orthobunyaviruses, WBV does not encode an NSs protein from the S segment. The NSs protein in most orthobunyaviruses is a non-essential protein during mammalian infection that plays a role in pathogenesis by shutting down host protein synthesis leading to cell death, and counteracting host antiviral responses [30–32]. The protein seems to be important for replication of Bunyamwera virus in mosquito cells [33].

Viruses from the Tete, Anopheles A and Anopheles B serogroups also do not express an NSs protein [34]. It has been proposed that these viruses lack an NSs protein, by assumption being unable to counteract mammalian host innate responses and thus being less virulent, and might be maintained in nature by an insect-only cycle through vertical (transovarial) and/or horizontal (venereal) transmission. If this is the case, these viruses bypass the need for generating the high viraemia in vertebrate hosts needed for transmission of arboviruses. It has been suggested that the NSs protein is a major factor in the zoonotic capacity of orthobunyaviruses, by allowing viruses expressing this protein to efficiently infect and replicate in vertebrates with a functioning innate type I interferon response [35]. However, Tacaiuma virus (TCMV) from the Anopheles A serogroup, has been shown to block interferon beta mRNA production despite lacking an ORF for NSs, suggesting that this virus has developed an alternative mechanism for counteracting the mammalian host innate response. Also, TCMV, which was originally isolated from a monkey and, subsequently, an *Anopheles* mosquito in South America, has been shown to cause febrile illness in humans and has been associated with other vertebrate hosts (bat, primate, bird and horse) [34, 36, 37]. The ability of WBV to counteract vertebrate host innate responses and cause high viraemia, despite the lack of NSs, needs future investigation through *in vitro* and *in vivo* studies. The decreased replication of WBV in HEK293 cells versus Vero cells might indicate that, without the expression of NSs, the virus is not able to replicate as efficiently in the presence of an interferon response, but this requires further in-depth investigation in *in vivo* interferon-competent versus interferon-deficient mouse models.

### Conclusion

Based on sequence divergence of the NP from other known orthobunyaviruses, it is likely that WBV represents a new species in the genus. Evidence of re-assortment of the M segment in two of the 11 isolates described here, which are quite divergent from the other nine isolates, likely indicates that the diversity of viruses in this proposed new species is higher than that indicated by the data. The high percentage of isolates from a rather low number of ectoparasite pools (11 isolates out of 273, amounting to 4 %) might indicate a very high prevalence in this specific bat colony, or may indicate that the timing of collection represents a period of high transmission rates. All isolates were made from ectoparasites collected during the dry winter period in South Africa when fruit is scarce, and also overlaps with the period when young adult bats have lost their maternal immunity. Aspects which need further investigation include whether WBV infects bats and what is the seroprevalence of bats collected from Mahlapitsi cave. Whether this new virus has any public or veterinary health importance is yet to be determined. Antibodies to the virus most closely related to WBV, KKV, have been detected in humans who suffered a mild disease [18]. The discovery of WBV expands the range of currently known orthobunyaviruses and also the arthropod host range. Its global distribution needs further investigation, considering the wide distribution of Egyptian fruit bats, the close association of bat flies with specific bat hosts, and the close association of arboviruses with their arthropod hosts. The ability of WBV to replicate *in vitro* in a mosquito cell line might indicate its ability to be disseminated by mosquitoes, but this needs further investigation through vector competence studies.

## Methods

### Virus source and isolation

Egyptian fruit bats (*Rousettus aegyptiacus*) were sampled between March 2013 and March 2014 at Mahlapitsi cave in the Mahlapitsi Valley, Limpopo province, South Africa, as described before [20]. Catch and release sampling included the collection and pooling of arthropods from parasitized bats into Eagle’s minimum essential medium (EMEM, Lonza). Pools of parasites were homogenized (30 Hz for 8 min by using a Tissuelyzer II and 5 mm stainless steel beads, Qiagen) and clarified supernatants used to inoculate Vero cell cultures. The wells of 24-well tissue culture plates (Nunc) were seeded with Vero E6 cells and grown to 80–90 % confluence in EMEM supplemented with antibiotics (100 U penicillin/ml 100 µg streptomycin/ml 250 ng amphotericinB/ml,Lonza) and 10 % fetal calf serum, at 37 °C and 5 % CO_2_. Culture medium was removed and the monolayers in individual wells inoculated with 200 µl of ectoparasite pool homogenates (one pool representing parasites from one bat). After 1 h adsorption at 37 °C, the inoculum was removed and fresh EMEM containing antibiotics and 2 % fetal calf serum added. Parasites were morphologically identified, and confirmed by amplification and amplicon sequencing of a region of the cytochrome oxidase 1 subunit gene, as belonging to the *Nycteribiidae* family, genus *Eucampsipoda* [20]. Inoculated cultures were monitored for development of CPEs for three blind passages of 14 days each. Cultures displaying CPEs were subjected to two or three more passages in 75 cm^2^ tissue culture flasks to prepare stocks. Stock virus titres were determined by standard TCID_50_ titrations on 96-well microtitre plates.

Bat fly homogenate pools and bat serum pools were subsequently tested by RT-PCR for the presence of WBV.

### Transmission electron microscopy

Processing of infected cell cultures for transmission electron microscopy was performed as described previously [20]. Briefly, culture supernatants from six of the 11 isolates were concentrated, adsorbed onto coated grids, negatively stained and viewed. Infected monolayers were routinely processed for ultramicrotomy (fixation, postfixation, ethanol dehydration, resin embedding, ultramicrotomy with double staining of 70 nm sections).

### Complete genome sequencing

Stock virus culture supernatant was added to Trizol-LS (Life Technologies) at a ratio of 100 µl supernatant to 300 µl Trizol-LS. RNA was extracted using a column based kit (Direct-Zol RNA kit, Zymo Research). To increase sensitivity, rRNA was depleted using the method described by Morlan *et al*. [38]. Samples were prepared for sequencing using the SISPA-RACE technique described previously [20]. Sequencing was performed either on an Illumina MiSeq or NextSeq 500 using either a 2×150 or 2×250 version2 kit. Illumina and SISPA adapter sequences were trimmed from the sequencing reads using Cutadapt-1.2.1 [39], quality filtering was conducted with Prinseq-lite [40] and reads were assembled into contigs using Ray Meta with kmer length=25 [41]. Resultant contigs were aligned to the NCBI sequence database using blast (www.ncbi.nlm.nih.gov/BLAST/).

### Phylogenetic and sequence analysis

The mega (version 6) program was used to prepare alignments (clustal w) of nucleic acid segment sequences, deduced amino acid sequences, phylogenetic trees and pairwise distance calculations [42]. The publicly available bunyavirus sequences used in the analysis were obtained from NCBI-Nucleotide (GenBank). Nucleotide sequences from a small number of viruses from each genus in the *Bunyaviridae* family were used to prepare a maximum-likelihood tree showing the placement of WBV in the family based on complete sequences of all three segments (L, M and S). Maximum-likelihood trees were prepared using amino acid sequences of all ORFs from all segments, showing the placement of WBV in the *Orthobunyavirus* genus relative to other viruses in this genus for which sequence is available on GenBank. Virus sequence accession numbers are summarised in Table 2. The evolutionary histories were inferred by using the maximum-likelihood method based on the Tamura–Nei model [43]. The trees with the highest log likelihood are shown. The percentage of trees in which the associated taxa clustered together is shown next to the branches. Initial tree(s) for the heuristic search were obtained by applying the neighbour-joining method to a matrix of pairwise distances estimated using the maximum-composite-likelihood approach. The trees are drawn to scale, with branch lengths measured in the number of substitutions per site. All positions containing gaps and missing data were eliminated. Evolutionary analyses were conducted in mega6 [42]. ORFs were located and deduced protein amino acid sequences prepared by using the CLC Genomics Workbench (Qiagen).

### Virus growth

*In vitro* replication of WBV was evaluated in three cell lines: Vero E6 (source: African green monkey kidney), HEK293 (source: human embryonic kidney) and C6-36 (source: *Aedes albopictus* mosquitoes). Cells were grown to 50–70 % confluence in 25 cm^2^ flasks, supernatant removed and respective flasks inoculated with 1 ml of 10^−1^, 10^−2^, 10^−3^ and 10^−5^ dilutions from stock virus (1×10^6.75^ TCID_50_ ml^1^ determined by standard 50 % tissue culture infectious doses titration) in EMEM. After 1 h adsorption at 37 °C (Vero E6 and HEK293) or 28 °C (C6-36), the inoculum was removed, cells washed with 5 ml PBS and fresh EMEM, antibiotics and 2 % fetal calf serum (Hyclone) added. Cultures were incubated for 13 days at 37 °C (Vero E6 and HEK293) or 28 °C (C6-36) while 0.5 ml aliquots of supernatant were collected from each flask directly after inoculation and addition of fresh medium (day 0), followed by days 1, 4, 5, 7, 11 and 13. RNA was extracted from 140 µl of the supernatant collections from respective days (QIamp viral RNA kit, Qiagen) and subjected to TaqMan real-time RT-PCR. A TaqMan real-time RT-PCR was developed to target the S segment of WBV. Primers and probe sequences are: forward MAOBV_783F (5′-TTGGCTTTCTTTGCATTCAG-3′), reverse MAOBV_869R (5′-ATGGTTTCAACCCTGAGGAA-3′) and probe MAOBV_831P (*FAM*-TCTTCACAAGTGGCAATGC-*BHQ*), with the number in the oligonucleotide name indicating the nucleic acid position in the S segment. Real-time RT-PCR was performed on the extracted RNA using the Qiagen One-step RT-PCR kit (Qiagen) on a SmartCycler (Cepheid) with the following parameters: reverse transcription (50 °C for 30 min), hot-start Taq activation (95 °C for 15 min) and 50 cycles of amplification (95 °C for 15 s; 52 °C for 25 s plus signal acquisition; 72 °C for 20 s). RNA extracted from diluted stock WBV (final 1×10^4.75^ TCID_50_ ml^1^) was used as a qualitative positive control in each run. A standard curve was prepared by testing a dilution series of stock virus (1×10^6.75^ TCID_50_ ml^1^) in triplicate and correlating *C*_t_ values to TCID_50_ equivalents.

## Supplementary Data

142 Supplementary File 1
