# Supplementary material for: Isolation of a novel orthobunyavirus from bat flies (Eucampsipoda africana)
Source: J Gen Virol. 2017 May 11;98(5):935–45. doi: 10.1099/jgv.0.000753 (PMC5656801; doi:10.1099/jgv.0.000753)
Supplement: Supplementary File 1 [file jgv-98-935-s001.pdf]

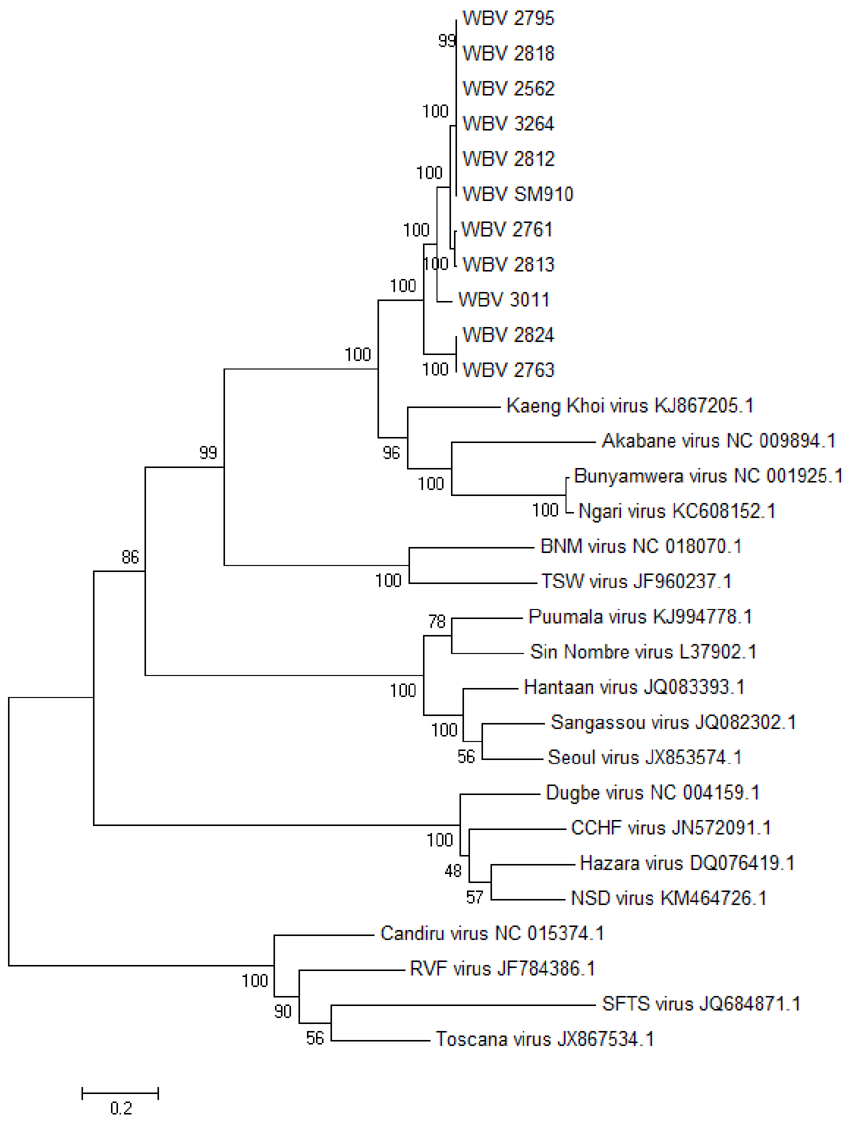

S1. Molecular phylogenetic analysis by the Maximum Likelihood method showing representative viruses from different genera in the *Bunyaviridae* family using large (L) segment nucleic acid sequences. Genbank accession numbers are indicated next to virus names (excluding WBV for which accession numbers are provided in Table 1).

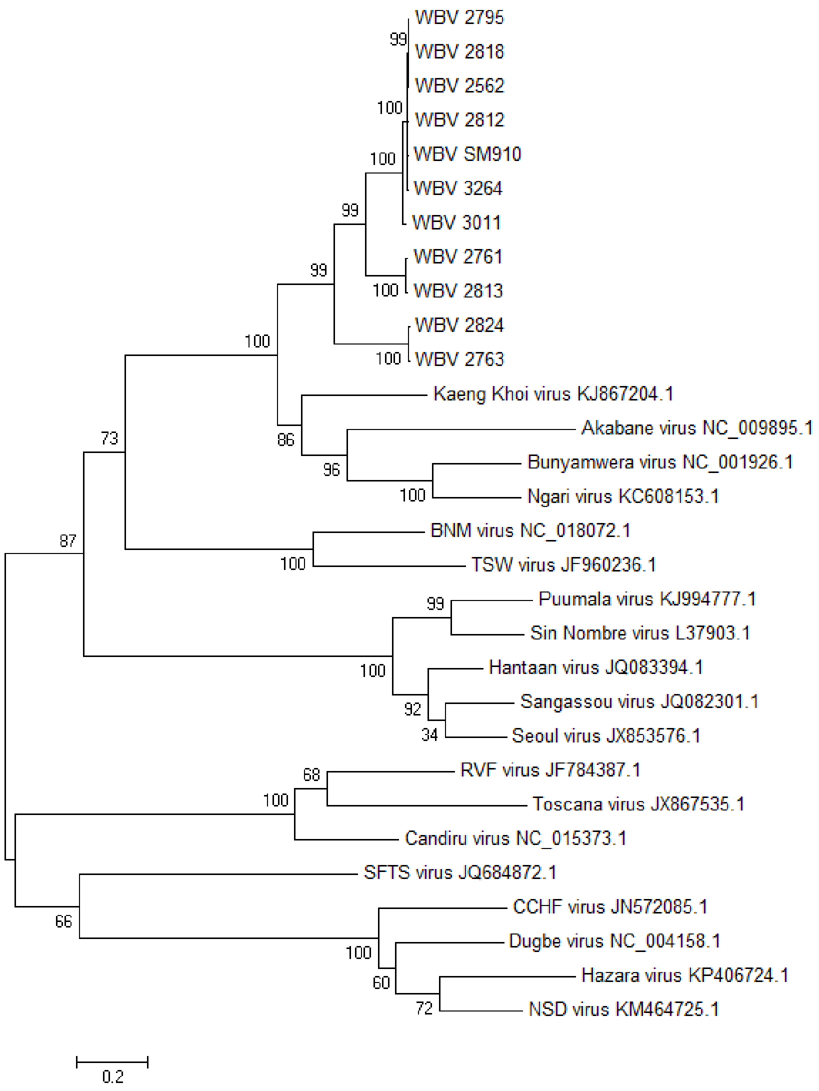

S2. Molecular phylogenetic analysis by the Maximum Likelihood method showing representative viruses from different genera in the *Bunyaviridae* family using medium (M) segment nucleic acid sequences. Genbank accession numbers are indicated next to virus names (excluding WBV for which accession numbers are provided in Table 1).

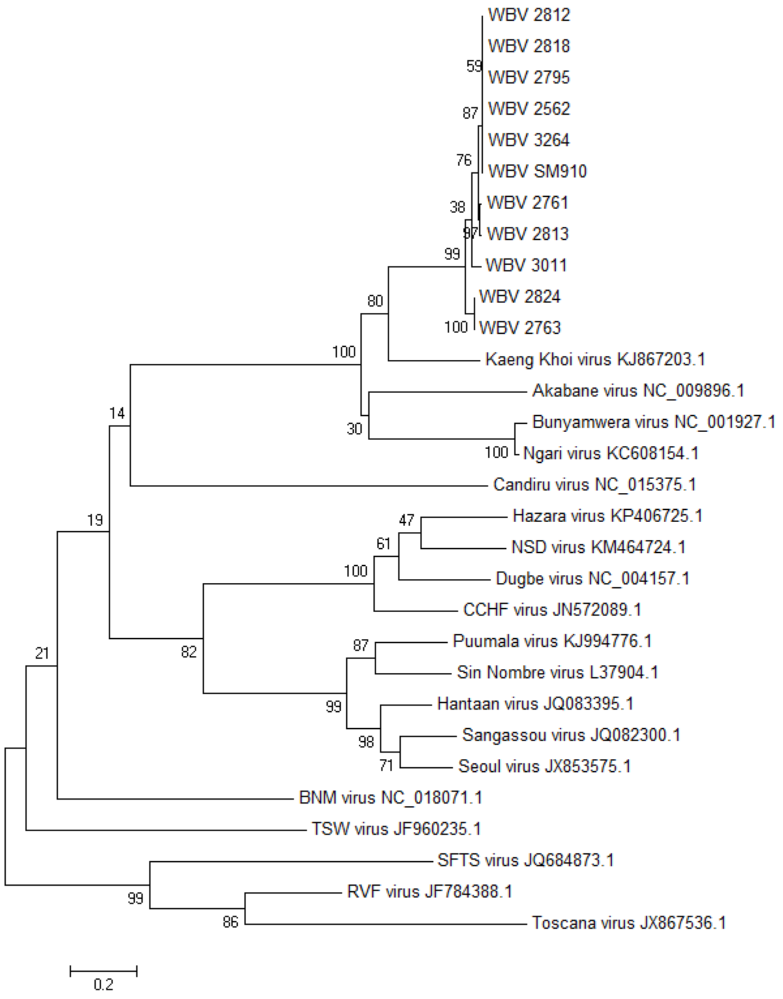

S3. Molecular phylogenetic analysis by the Maximum Likelihood method showing representative viruses from different genera in the *Bunyaviridae* family using small (S) segment nucleic acid sequences. Genbank accession numbers are indicated next to virus names (excluding WBV for which accession numbers are provided in Table 1).
